# Egg Characteristics of Female Common Terns Are Repeatable, and Vary With Maternal Age and Laying Order

**DOI:** 10.1002/ece3.72455

**Published:** 2025-11-19

**Authors:** Coraline Bichet, Maria Moiron, Nathalie Kürten, Oscar Vedder, Sandra Bouwhuis

**Affiliations:** ^1^ Centre d'Etudes Biologiques de Chizé, UMR‐CNRS 7372 La Rochelle Université Villiers‐en‐Bois France; ^2^ Institute of Avian Research Wilhelmshaven Germany; ^3^ Department of Evolutionary Biology Bielefeld University Bielefeld Germany

**Keywords:** egg camouflage, egg coloration, egg elongation, egg pointedness, egg quality, egg shape, egg volume, polar asymmetry, spottiness

## Abstract

Avian eggs exhibit striking variability in size, shape, colour, and maculation, not only among but also within species. Technical and analytical advances in image analysis offer the opportunity to understand the factors underpinning this variability, especially when individual‐based longitudinal data are available. Making use of such data, collected over four years, we investigated sources of variation in eight egg characteristics capturing the colour, spottiness, shape, and size of 1589 eggs from 687 clutches produced by 330 female common terns ( *Sterna hirundo* ) of known age. We found a high repeatability of the eight egg traits, both within clutches (range 0.48–0.77), and among clutches of the same female laid in different years (range 0.48–0.73). We also observed a within‐female increase in egg size and spottiness with age, and evidence for selective disappearance of females producing spottier eggs, suggesting that egg maculation could reveal female quality. We also found that the size and shape of eggs were affected by their laying order within the clutch, suggesting that these traits may mediate intra‐brood competition. We suggest further studies to identify the specific agents of selection that shape variation in egg size and morphology, to fully understand the eco‐evolutionary significance of this extended female phenotype and its potential consequences for reproductive success and offspring survival.

## Graphical abstract

We investigated sources of variation in eight egg characteristics capturing the colour, spottiness, shape, and size of 1589 eggs from 687 clutches produced by 330 female common terns ( *Sterna hirundo* ) of known age. We found a high repeatability of the eight egg traits, both within clutches and among clutches of the same female. We also observed a within‐female increase in egg size and spottiness with age, and selective disappearance of females producing spottier eggs, while the size and shape of the eggs were affected by the laying order.

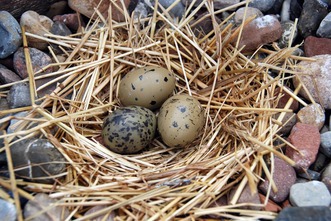

## 1 Introduction

Avian eggs exhibit striking inter‐specific variability in the colour and maculation of their shells as well as their shape and size (Kilner 2006; Cassey et al. 2012; Hanley et al. 2015; Stoddard et al. 2017; Birkhead et al. 2018). These egg characteristics, however, do not only vary among species, but within species as well (Birkhead, Thompson, and Biggins 2017; Ducay et al. 2021, 202; Hauber et al. 2019; Holveck et al. 2017). Technical and analytical advances, combined with the availability of long‐term individual‐based studies, now offer the opportunity to deepen our understanding of factors explaining this variation.

One important aspect is the extent to which the colour, maculation, size and shape of eggs (hereafter referred to as “egg characteristics”) can vary within females. If intra‐individual variation in egg characteristics is large, this would imply that these characteristics are mainly determined by varying individual factors (e.g., age, condition, epigenetics) and/or environmental factors (e.g., abiotic conditions, food availability). If, on the other hand, intra‐individual variation would be low, egg characteristics could be mainly driven by stable individual characteristics that are genetically heritable or otherwise determined early in life (McCormack and Berg 2010), and/or stable environmental factors (e.g., micro‐habitat effects). Elucidating this requires longitudinal studies of the characteristics of the eggs laid by the same female. Various studies have investigated the consistency of egg characteristics within females or clutches, with some reporting a high individual repeatability in both colour and shape indices (Birkhead, Thompson, and Biggins 2017; Corti et al. 2018; Dearborn et al. 2012; Gómez‐Bahamón et al. 2023; Hauber et al. 2019; Sulc et al. 2022, see Schmitz Ornés et al. 2023 for a meta‐analysis), while others found more variability, for example related to female fitness proxies (such as body mass, immune parameters, oxidative status; reviewed by Reynolds et al. 2009).

With respect to variation in eggshell colouration, blue‐green hues are produced by the pigment biliverdin IXα, while brownish hues and spots are produced by protoporphyrin IX (Gorchein et al. 2009; Kennedy and Vevers 1976). Both pigments are derived from a common precursor (Wang et al. 2009) and produced in the shell gland during the biosynthesis of blood (Zhao et al. 2006). Biliverdin is considered to be able to scavenge free radicals and therefore to have antioxidant properties (Duval et al. 2013; McDonagh 2001), reducing damage arising from oxidative stress and/or during immune responses (Baylor and Butler 2019; Kaur et al. 2003). Protoporphyrin, on the other hand, is considered to be a prooxidant (Duval et al. 2013), able to cause oxidative stress in the liver (Shan et al. 2000). As such, variation in eggshell colouration is likely to be related to female variation in antioxidant and immune capacities (Morales et al. 2006; Moreno and Osorno 2003; Reynolds et al. 2009), although the nature of this relationship has been debated. On the one hand, only females with high antioxidant/immune capacities or oxidative tolerance (i.e., high‐quality females) might be able to allocate a high level of biliverdin and protoporphyrin to their eggshells (Reynolds et al. 2009; Moreno and Osorno 2003; Corti et al. 2018). On the other hand, with respect to protoporphyrin, high levels of this pigment may be deposited into the eggshells by females in poor condition, or with a high stress level, if deposition is a passive process (Martínez‐de la Puente et al. 2007). As a separate line of investigation, studies have found correlations between eggshell colour or maculation and female contamination levels (Hargitai, Nagy, Nyiri, et al. 2016; Jagannath et al. 2008), which could also suggest egg colour and maculation to be indicative of female “quality” (*sensu* Wilson and Nussey 2010). Besides being a potential indicator of female quality, egg colouration and maculation can signal shell structure and strength (Bulla et al. 2012; Cherry and Gosler 2010; Gosler et al. 2011; Gosler et al. 2005), and/or play a role in thermoregulation (Wisocki et al. 2020), water loss (Higham and Gosler 2006), protection against UV (Maurer et al. 2011) and antimicrobial defence (Ishikawa et al. 2010; Zhu et al. 2010). Moreover, as eggshells can be perceived by various observers, their colour and maculation are likely involved in camouflage, in order to avoid egg predation (Stoddard et al. 2011; Westmoreland 2008; Westmoreland and Kiltie 2007), in recognition of clutches from conspecifics (Holveck et al. 2017; Quach et al. 2021), and in the avoidance of brood parasitism (Lyon 2007, 2003; Lyon and Eadie 2008).

With respect to egg shape, studies at the intra‐specific level are rare (mostly focused on eggs of cliff‐nesting guillemots— *Uria aalge* and *Uria lomvia* —Smith 1966; Birkhead, Thompson, and Biggins 2017; Birkhead, Thompson, Jackson, et al. 2017; Birkhead et al. 2018, but see Encabo et al. 2002; Ojanen et al. 1978 for passerine species), but seem to indicate that egg shape could also be linked to female quality, when correlated with shell resistance (Birkhead et al. 2018; Smith 1966). Most studies, however, have focused solely on egg size and concluded that the traits explaining its intraspecific variation vary among species (reviewed in Christians 2002). In general, however, egg size is found to correlate with female quality, with females in better condition being able to produce larger eggs, and these larger eggs producing larger, or otherwise more viable, chicks (Nisbet 1978; Reid and Boersma 1990; Christians 2002; Krist 2011; Pick et al. 2016; Vedder et al. 2023). However, how females vary the size of their eggs—with or without changing their shape—and which specific shape components are most likely to vary, is poorly understood.

If egg characteristics are related to female “quality,” these egg characteristics are expected to vary with female age, as individual performance typically changes with age in birds (reviewed in Bouwhuis and Vedder 2017). Studies that investigated how egg characteristics vary with female age indeed, for instance, found that older females produce eggs that are shorter, broader, and larger (Coulson 1963), but also whiter (Hodges et al. 2020), or more colourful (Siefferman et al. 2006). Only when studies are longitudinal, and characterize the colour, maculation, size, and shape of multiple eggs laid at different ages by the same female, however, can they understand the origin of such age effects, and disentangle any within‐individual effect of age from between‐individual processes, such as selective disappearance, which would occur if females laying eggs with certain characteristics would also exhibit traits that cause them to die younger (i.e., be less common in older age classes) (van de Pol and Verhulst 2006; van de Pol and Wright 2009). The latter may be expected for egg characteristics that are indicative of female quality.

Egg characteristics can also vary with the laying order of eggs in a clutch. Studies in tree sparrows ( *Passer montanus* ) and barn swallows ( *Hirundo rustica erythrogaster* ), for instance, found that last‐laid eggs were less pigmented (Poláček et al. 2017) and less maculated (Beech et al. 2022), suggesting that the quantity of the pigments a female provides to her eggs is limited. Similarly, another study on russet sparrows (*Passer cinnamomeus*) found that last‐laid eggs are whiter (Huo et al. 2018). However, results from a long‐term study on great tits ( *Parus major* ) showed maculation to increase with laying order, which was interpreted to provide structural compensation for a decrease in shell thickness with laying order (Gosler 2006). In seabirds, it is frequently observed that egg size decreases with laying order (Bollinger 1994; Garcia et al. 2011; Vedder, Zhang, and Bouwhuis 2017) and is assumed that producing a small final egg may facilitate the rapid mortality of excess offspring when food is limited (“brood reduction strategy,” Slagsvold et al. 1984). Other studies, mainly in passerine birds with large clutches (Socias‐Martínez et al. 2025), however, reported an increase in egg volume with laying order (“brood survival strategy,” Clark and Wilson 1981).

Here we report on a large‐scale study of the colouration, maculation, shape, and size of 1589 eggs, from 687 clutches, produced by 330 known‐age common tern ( *Sterna hirundo* ) females between 2017 and 2020. Using image analyses from digitized pictures taken in a standardized purpose‐built box, we assess the existence of (co)variance among eight egg characteristics, quantify the female repeatability of these characteristics, study how they change as females age, and test for laying order effects. If egg characteristics are mainly driven by stable female traits, we expect these characteristics to be repeatable within females. Moreover, if eggs of a certain appearance are costly to produce, we expect egg characteristics to depend on female quality, and therefore, to be correlated with female age. This correlation could either take hold within individuals, as the quality of females may vary with age, or between individuals, when better quality females are overrepresented within older age classes. With respect to laying order, we expect effects on egg characteristics that predominantly have an effect on the chicks after hatching and thereby adaptively mediate the intra‐brood competition between siblings.

## 2 Materials and Methods

### 2.1 Species and Study Population

This study is part of a long‐term individual‐based study of common terns from a mono‐specific breeding colony located at the Banter See in Wilhelmshaven, on the German North Sea coast (53°30′40″ N, 08°06′20″ E). Common terns are socially and genetically monogamous (Gonzalez‐Solis et al. 2001; Griggio et al. 2004) migratory seabirds that arrive at the Banter See colony site in early spring (Becker and Ludwigs 2004; Kürten et al. 2022). The large‐scale individual‐based study started in 1992, when 101 breeders and all fledglings were individually marked with subcutaneous transponders (model ID 100; TROVAN, Germany) (Becker and Wendeln 1997). Since then, all local fledglings have been marked with such transponders each year (Becker et al. 2001). The sex of each transpondered bird has been determined by standard molecular methods (Becker and Wink 2003).

The colony site consists of six concrete islands (10.7 × 4.6 m each) surrounded by 60 cm high walls supporting 44 platforms for the terns to land on and rest. All islands are covered by a uniform type of river gravel, on which the terns lay their eggs. Clutch size varies between one and three eggs, which are laid, and hatch, at 1–2 day intervals (Becker and Ludwigs 2004; Vedder et al. 2019). During three‐weekly checks, all new eggs are measured (greatest length and breadth) and their laying order is recorded. For 79% of the eggs included in our study, laying order was known exactly, whereas for the remaining 21% of eggs, it was assigned randomly upon encountering two new eggs on the same day. The random assignment with respect to egg characteristics should mean that although true effects may be underestimated, there is no reason to expect false positives.

Transponder‐marked breeders were identified by placing an antenna around each nest for at least 24 h during incubation, which is shared by the two partners. Given that brood parasitism is rare in our colony (3%, unpublished data), eggs can be highly reliably assigned to their parents. Egg predation is rare as well, since the colony is completely protected against ground predators, but carrion crows ( *Corvus corone* ) occasionally take eggs.

### 2.2 Egg Pictures

While failed clutches may be replaced (Becker and Zhang 2011; Wendeln et al. 2000), second clutches are very rare (Becker and Ludwigs 2004) and only produced under particular conditions (Moore and Morris 2005). As such, we focused our analyses on the eggs of first clutches only. Between 2017 and 2020, all eggs of first clutches of females of known identity and age were temporarily removed and replaced by dummy eggs. They were then transported to an on‐site field station using a shockproof box and photographed in a box (40.8 × 40.8 × 45.5 cm) custom‐built for this purpose. To ensure standardisation, the inside of the box was lit with a constant circular LED strip (1500 lm), and all pictures were taken using constant parameters (manual mode, exposure 1/40, aperture F10, ISO 200, WB 40000K) set on a reflex camera (Canon 400D with 18–55 mm Canon lens) fixed at the top of the box. Each egg was placed on the pedestal fixed inside the box and, in the case of 1430 eggs, photographed twice, with an egg turn in between. In 159 cases where eggs were partly stained with soil or faeces, only a single picture from the clean side was taken. In total, we therefore took 3019 pictures of 1589 eggs from 687 clutches laid by 330 females (Figure S1). Of these 330 females, 129 had their eggs photographed in one year, while 89, 68, and 44 had their eggs photographed in two, three, and all four years, respectively.

### 2.3 Image Analyses

All image analyses were conducted by a single experimenter (CB).

#### 2.3.1 Egg Spottiness

We used the plug‐in “Threshold colour” of the software ImageJ (v1.54d, Schneider et al. 2012) to adjust the colour parameters in each picture until the spots were discriminated from the shell matrix (see Holveck et al. 2012 for a similar method) and the number of pixels making up these spots could be measured. The colour parameters were then adjusted to obtain the number of pixels of the full egg surface, allowing us to calculate the spottiness by dividing the pixels of the spotted surface by those of the total eggshell surface. The more the eggs are covered by spots, the higher the quantity of the pigments dedicated to spots, and the higher the spottiness value.

#### 2.3.2 Eggshell Colouration

Also using ImageJ, the RGB (Red, Green, Blue) colour space of the eggshell (without the spots, see Section 1) was extracted from each picture. Using the “Wand‐Tool” plug‐in (mode: 4‐connected, tolerance: 2), RGB values were extracted from five randomly selected areas (see Holveck et al. 2012 for a similar method) and the HSV (Hue, Saturation, Value) colour space was obtained from these RGB values using the R (version 3.6.1, R Core Team 2014) function “rgb2hsv.” Hue (H) describes the dominant wavelength of the colour, and varies from 0 (red) to 0.36 (magenta). Saturation (S) is the amount of the dominant colour (H) in the colour, and varies from 0 (for grey) to 1 (for a primary colour). Value (V) represents the amount of light in the colour (i.e., the brightness), and also varies from 0 (dark) to 1 (light). The HSV colour space has been widely used across taxa to describe colouration in wild populations (Stevens et al. 2007, 2009), and has been shown to correlate with egg pigment concentrations in bird eggs (Poláček et al. 2017).

#### 2.3.3 Egg Size and Shape

Egg shape can be characterized by many parameters, and which the most appropriate ones are is still under debate (Biggins et al. 2022). We decided to follow the recommendations provided by Birkhead et al. (2019), and used three parameters, derived from Preston's method (Biggins et al. 2022; Preston 1968; Preston 1953), to describe egg shape: pointedness, elongation, and polar asymmetry. These parameters were calculated from the egg pictures using the R script provided by Biggins et al. (2018) and the R package “EBImage” (Pau et al. 2010). Egg volume (in mm^3^) was calculated according to the R script and the equation provided by Biggins et al. (2018).

For the eggs that were photographed twice, we calculated the means of the egg characteristics obtained from the pictures. In addition, we tested the “measurement repeatability” of the two measures obtained per egg, and found it to be very high in all cases, ranging between 0.909 and 0.995 (Table S1).

### 2.4 Statistical Analyses

First, we tested the Pearson's correlations among the eight egg characteristics: spottiness, Hue, Saturation, Value, volume, pointedness, elongation, and polar asymmetry (all modelled with a Gaussian error distribution). Second, we conducted a principal component analysis (PCA) to assess how the eight egg characteristics loaded onto fewer axes of variation (Tables S2 and S3). The PCA was run using the R (version 3.6.1, R Core Team 2014) function “prcomp” and visualized using the R packages “factoextra” (Kassambara and Mundt 2020) and “FactoMineR” (Lê et al. 2008).

Third, to test whether the eight egg characteristics varied with female age and differed between eggs of different laying order and from differently sized clutches, we ran a series of eight linear mixed models (LMMs) using the function “lmer,” implemented in the R package “lme4” (Bates et al. 2015). The females' actual age was partitioned into an “average age” and “delta age” component (following van de Pol and Wright 2009) and both variables were added as explanatory variables. Average age was calculated as the average of all ages at which we measured a female's egg parameters, whereas delta age was calculated as the difference between the female's actual age at measurement and her average age (i.e., delta age = age − average age). When modelled jointly, average age estimates the among‐individual age effect, and delta age the within‐individual age effect (van de Pol and Wright 2009). Should these effects differ significantly from one another, this would mean that the effect of age among females cannot be explained by changes with age within females, indicating that there is selective (dis)appearance of females with certain egg parameters from the older age classes (van de Pol and Wright 2009). Since the effects of laying order may not be independent from, or not identifiable when not also accounting for, clutch size, we created a six‐level categorical variable (“CS.LO”) representing a combination of clutch size (CS: 1, 2 or 3 eggs) and laying order (LO: egg laid 1st, 2nd or 3rd) (also see Vedder, Zhang, and Bouwhuis 2017; Vedder et al. 2019) and added it to each model. Female identity, clutch identity (nested in female identity), and year were added as random effects to account for the fact that eggs laid by the same female, in the same nest by the same female or in the same year are not independent from each other. Additionally, we checked whether there was a linear change in egg characteristics across the four years by fitting year as a continuous fixed effect in our models. We did not find any significant linear trends for any of the eight egg characteristics and hence did not include year as a fixed effect in the final models.

We calculated the repeatability of the eight egg characteristics (i) at the female level including egg data for all clutches produced across the years and (ii) at the clutch level including those eggs produced in the same clutch in a given year. Female and female‐within‐clutch repeatability estimates could be considered as “long‐term” and “short‐term” repeatability estimates, respectively (*sensu* Araya‐Ajoy et al. 2015). Female repeatability is calculated as the variance explained by female identity divided by the total variance not attributable to fixed effects (Nakagawa and Schielzeth 2013); while female‐within‐clutch repeatability is calculated as the variance explained by female identity, plus the variance explained by clutch identity (nested in female identity), divided by the total variance not attributable to fixed effects. As such, female‐within‐clutch repeatability must be equal to, or higher than female repeatability.

We used the “sim” function from the R‐package “arm” to simulate values from the posterior distributions of the model parameters (Gelman and Su 2024). Based on 5000 simulations, we extracted 95% credible intervals (CI) around the mean of each fixed and random effect. Model assumptions were tested by checking the residuals' independence, normality and homogeneity, and autocorrelation. Assessment of statistical support was obtained from the posterior distribution of each parameter. We also provide *p* values to test the significance of fixed effects (*α* = 0.05).

Finally, using an ANOVA, we also tested the significance (*α* = 0.05) of the “CS.LO” effect on each egg parameter and conducted a posteriori tests (Tukey HSD) to assess the differences between two “CS.LO” categories. For this, we focused on differences between the laying order of eggs within a clutch (i.e., eggs 2‐1 compared to 2‐2 and eggs 3‐1 compared to 3‐2 and 3‐3).

## 3 Results

**FIGURE 1:**
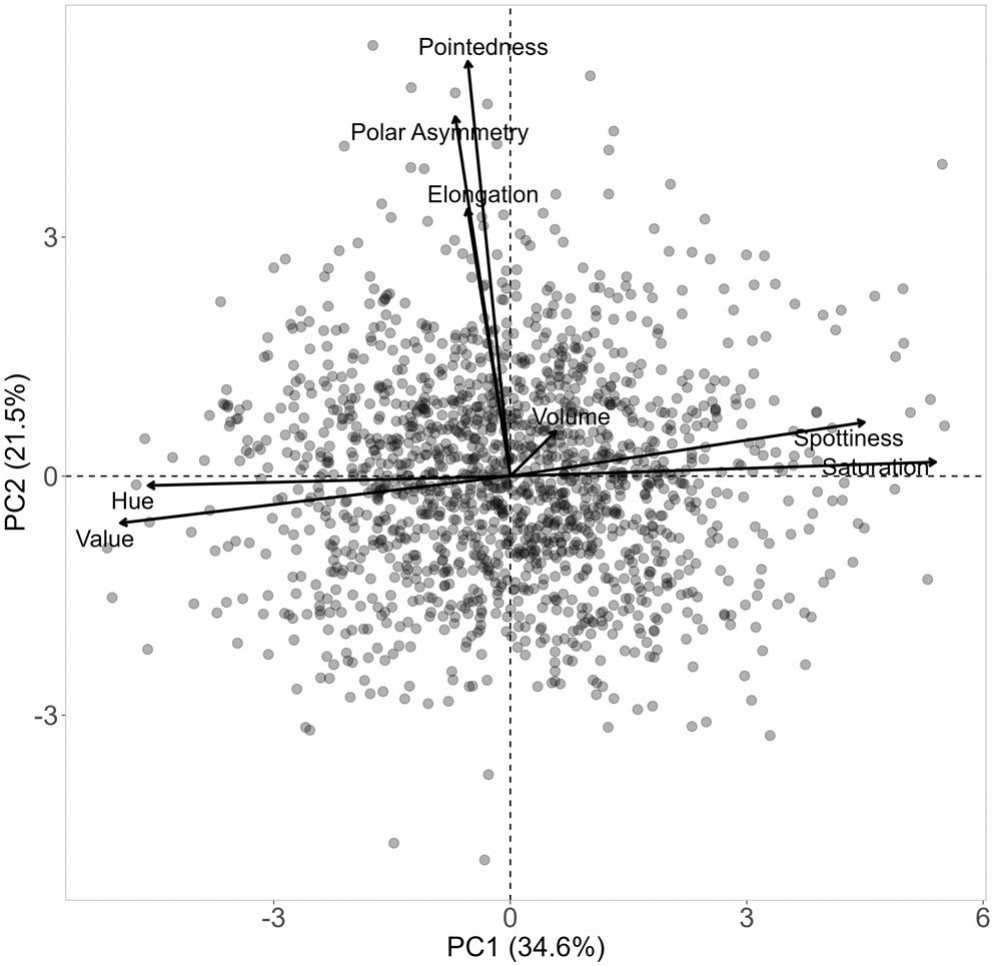
Results from a principal component analysis of eight egg characteristics (spottiness, Hue, Saturation, Value, volume, pointedness, elongation, polar asymmetry) of 1589 eggs (grey circles) from 687 clutches laid by 330 female common terns between 2017 and 2020. The arrows represent the projection of the eight characteristics onto the first two PCA axes (PC1 and PC2), which explained 34.6% and 21.5% of the variation, respectively (also see Tables S2 and S3).

The mean (±SE), minimum and maximum, as well as the distribution of all eight egg characteristics assessed (spottiness, Hue, Saturation, Value, volume, pointedness, elongation, polar asymmetry) are reported and visualized in Table 1. The four egg characteristics related to colour and maculation (spottiness, Hue, Saturation, Value) were correlated with each other, as were those describing size and shape (volume, pointedness, elongation, and polar asymmetry) (Figure S2). The PCA showed that the first two axes could explain 34.6% and 21.5% of the variation, respectively (Figure 1 and Table S2), with colour/maculation indices having high loading values for the first axis (negatively for Hue and Value, positively for Saturation and spottiness), and shape/size indices having high loading values for the second axis (positively for all indices: volume, pointedness, elongation, and polar asymmetry). This means that bluer/greener eggs were also brighter, but less spotty and saturated; and that large eggs were also pointy, long, and asymmetric.

**TABLE 1:** Descriptive statistics for, and distributions of, eight egg characteristics measured for 1589 eggs laid in 687 clutches by 330 female common terns between 2017 and 2020.

Egg parameter	Mean ± SD	Minimum	Maximum	Distribution
Spottiness	0.236 ± 9.323 × 10^−2^	0.006 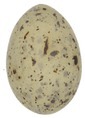	0.776 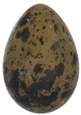	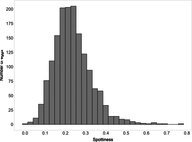
Hue	0.136 ± 1.693 × 10^−2^	0.089 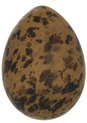	0.206 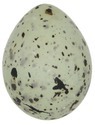	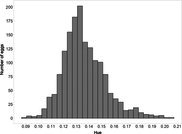
Saturation	0.359 ± 7.584 × 10^−2^	0.133 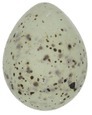	0.549 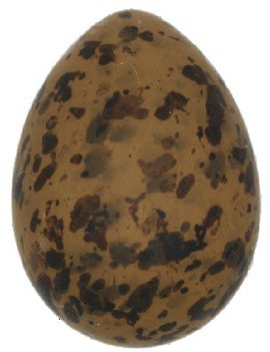	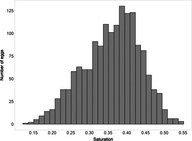
Value	0.788 ± 6.668 × 10^−2^	0.538 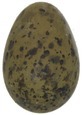	0.981 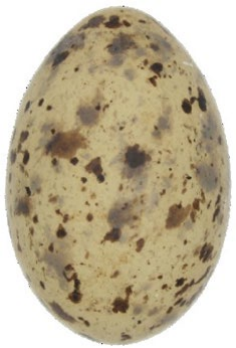	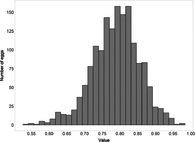
Volume (mm^3^)	18.872 ± 1.466	9.642 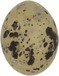	23.548 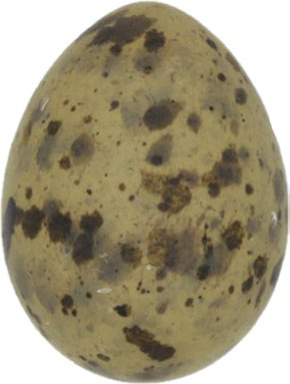	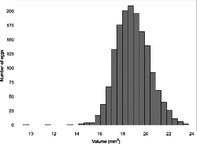
Pointedness	0.566 ± 1.478 × 10^−2^	0.510 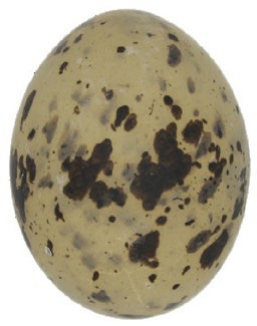	0.633 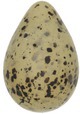	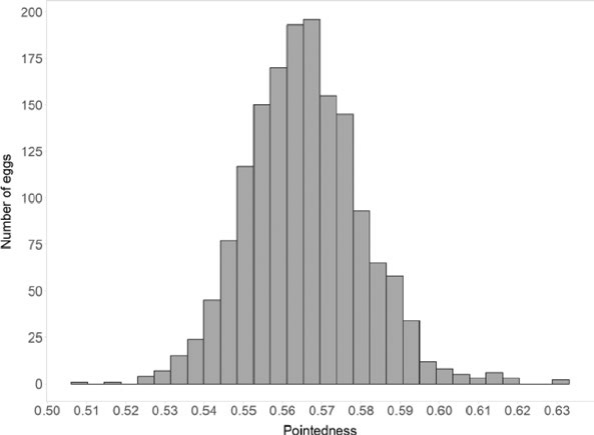
Elongation	1.383 ± 6.241 × 10^−2^	1.210 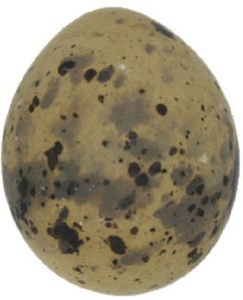	1.640 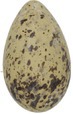	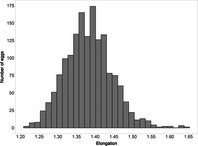
Polar asymmetry	2.258 ± 4.544 × 10^−1^	1.148 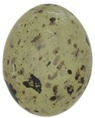	4.015 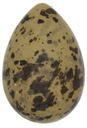	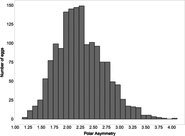

**FIGURE 2:**
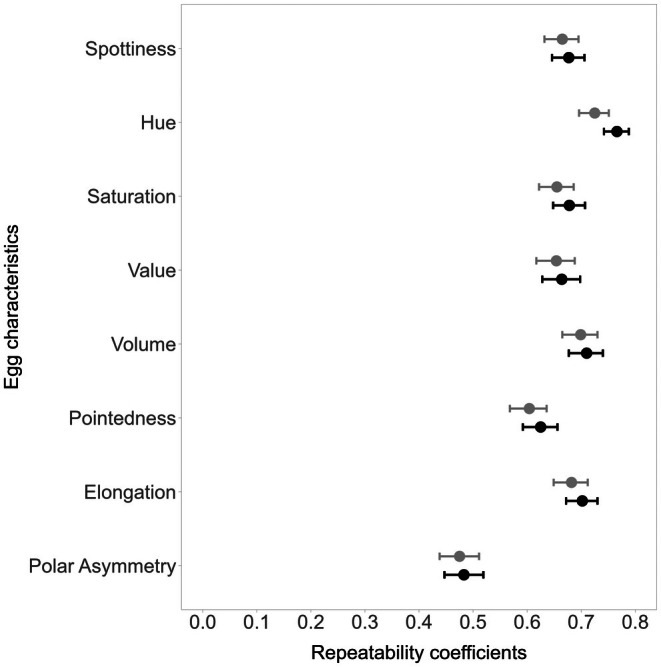
Estimates of the repeatability of eight egg characteristics for female common terns across years (grey circles) and within clutches (black circles), with their 95% credible intervals (bars), assessed using 1589 eggs from 687 clutches laid by 330 individual female common terns in the years 2017–2020.

The characteristics of all eggs produced by the same female across the years were moderately to highly repeatable, with female repeatability coefficients varying from 0.48 for polar asymmetry to 0.73 for Hue (Figure 2 and Table S4), meaning that individual females lay similar eggs in their different clutches produced in different years. Eggs from the same clutch were equally or only slightly more similar than eggs from different clutches of the same female among years, with female‐within‐clutch repeatability coefficients varying from 0.48 for polar asymmetry to 0.77 for Hue (Figure 2 and Table S4).

**FIGURE 3:**
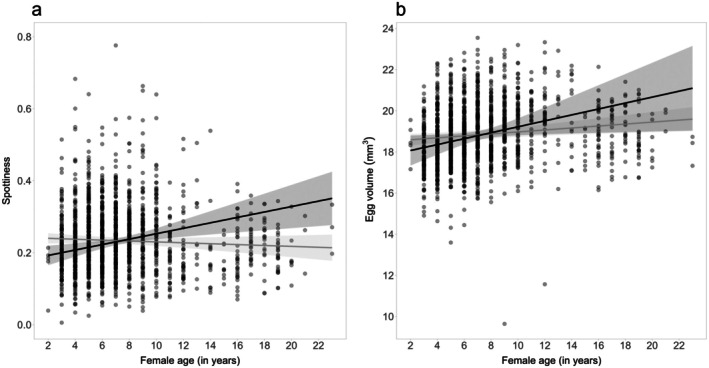
Relationship between female age and (a) eggshell spottiness and (b) egg volume. Dots represent the values for 1589 eggs from 687 clutches laid by 330 female common terns, solid lines the model predictions with their standard errors (grey areas). The black lines represent the within‐female effect, while the grey lines represent the among‐female effect.

Regarding the within‐female effect of age, we found eggs to become spottier as females grew older (*β* = 7.55 × 10^−3^, 95% CI = 2.91 × 10^−3^–1.22 × 10^−3^, Figure 3a and Table 2). In addition, there was a difference between the effects of average age and delta age for egg spottiness (*β* = 8.79 × 10^−3^, 95% CI = 3.51 × 10^−3^–1.41 × 10^−2^), indicating that females producing spottier eggs were less likely to be found breeding at older ages (suggesting selective disappearance of females producing spottier eggs, Figure 3a and Table 2). Egg volume also tended to increase with age within females (*β* = 1.41 × 10^−1^, 95% CI = 1.56 × 10^−2^–2.76 × 10^−1^, Figure 3b and Table 2), but for this trait there was no indication of selective disappearance of females with a certain egg size (*β* = 9.66 × 10^−2^, 95% CI = −4 × 10 × 10^−2^–3.36 × 10^−1^, Figure 3b and Table 2).

**TABLE 2:** Results from general linear mixed models testing whether variation in the spottiness, Hue, Saturation, Value, volume, pointedness, elongation or polar asymmetry, of 1589 eggs laid in 687 clutches by 330 female common terns between 2017 and 2020 is explained by among‐ (average age) and within‐individual (delta age) components of female age, and/or the combination of clutch size and laying order (CS.LO with 1.1 as a reference category).

Egg parameter	Spottiness			Hue			Saturation			Value		
Fixed effect	Mean	95% CI	*p*	Mean	95% CI	*p*	Mean	95% CI	*p*	Mean	95% CI	*p*
Intercept	**2.28 × 10** ^**−1**^	**1.99 × 10** ^**−1**^, **2.56 × 10** ^**−1**^	**< 0.001**	**1.28 × 10** ^**−1**^	**1.23 × 10** ^**−1**^, **1.33 × 10** ^**−1**^	**< 0.001**	**3.66 × 10** ^**−1**^	**3.43 × 10** ^**−1**^, **3.90 × 10** ^**−1**^	**< 0.001**	**7.85 × 10** ^**−1**^	**7.63 × 10** ^**−1**^, **8.06 × 10** ^**−1**^	**< 0.001**
Average age	−1.24 × 10^−3^	−3.46 × 10^−3^, 9.47 × 10^−4^	0.270	**7.47 × 10** ^**−4**^	**3.35 × 10** ^**−4**^, **1.16 × 10** ^**−3**^	**< 0.001**	−1.20 × 10^−3^	−2.97 × 10^−3^, 6.08 × 10^−4^	0.188	−2.51 × 10^−4^	−1.93 × 10^−3^, 1.45 × 10^−3^	0.769
Delta age	**7.55 × 10** ^**−3**^	**2.91 × 10** ^**−3**^, **1.22 × 10** ^**−2**^	**0.019**	3.37 × 10^−4^	−3.63 × 10^−4^, 1.04 × 10^−3^	0.346	−1.11 × 10^−3^	−5.59 × 10^−3^, 3.27 × 10^−3^	0.636	−4.54 × 10^−3^	−1.02 × 10^−2^, 1.33 × 10^−3^	0.166
CS.LO(2‐1)	6.02 × 10^−3^	−2.04 × 10^−2^, 3.28 × 10^−2^	0.654	2.87 × 10^−3^	−1.43 × 10^−3^, 7.16 × 10^−3^	0.190	−4.06 × 10^−3^	−2.50 × 10^−2^, 1.63 × 10^−2^	0.710	1.02 × 10^−2^	−6.51 × 10^−3^, 2.70 × 10^−2^	0.242
CS.LO(2‐2)	1.56 × 10^−2^	−1.13 × 10^−2^, 4.22 × 10^−2^	0.246	2.29 × 10^−3^	−1.92 × 10^−3^, 6.64 × 10^−3^	0.295	5.13 × 10^−3^	−1.55 × 10^−2^, 2.54 × 10^−2^	0.638	4.11 × 10^−3^	−1.26 × 10^−2^, 2 × 10 × 10^−2^	0.637
CS.LO(3‐1)	1.73 × 10^−2^	−9.09 × 10^−3^, 4.37 × 10^−2^	0.191	3.09 × 10^−3^	−1.09 × 10^−3^, 7.27 × 10^−3^	0.152	−2.82 × 10^−3^	−2.33 × 10^−2^, 1.77 × 10^−2^	0.794	3.21 × 10^−3^	−1.36 × 10^−2^, 1.98 × 10^−2^	0.710
CS.LO(3‐2)	1.98 × 10^−2^	−6.36 × 10^−3^, 4.57 × 10^−2^	0.135	**5.20 × 10** ^**−3**^	**9.93 × 10** ^**−4**^, **9.29 × 10** ^**−3**^	**0.016**	−3.74 × 10^−3^	−2.45 × 10^−2^, 1.68 × 10^−2^	0.728	5.74 × 10^−3^	−1.07 × 10^−2^, 2.22 × 10^−2^	0.504
CS.LO(3‐3)	1.44 × 10^−2^	−1.19 × 10^−2^, 4.03 × 10^−2^	0.274	2.68 × 10^−3^	−1.54 × 10^−3^, 6.83 × 10^−3^	0.213	7.77 × 10^−3^	−1.30 × 10^−2^, 2.83 × 10^−2^	0.471	1.60 × 10^−2^	−4.27 × 10^−4^, 3.24 × 10^−2^	0.063
Difference between average age and delta age	**8.79 × 10** ^**−3**^	**3.51 × 10** ^**−3**^, **1.41 × 10** ^**−2**^		−4.09 × 10^−4^	−1.22 × 10^−3^, 4.01 × 10^−4^		9.57 × 10^−5^	−4.83 × 10^−3^, 4.89 × 10^−3^		−4.24 × 10^−3^	−1.04 × 10^−2^, 2.09 × 10^−3^	

Random effect	Mean	95% CI		Mean	95% CI		Mean	95% CI		Mean	95% CI	
Female identity	7.39 × 10^−3^	6.37 × 10^−3^, 8.48 × 10^−3^		2.40 × 10^−4^	2.09 × 10^−4^, 2.73 × 10^−4^		4.66 × 10^−3^	4.04 × 10^−3^, 5.33 × 10^−3^		4.56 × 10^−3^	3.94 × 10^−3^, 5.22 × 10^−3^	
Clutch identity	1.36 × 10^−4^	1.19 × 10^−4^, 1.56 × 10^−4^		1.30 × 10^−5^	1.18 × 10^−5^, 1.52 × 10^−5^		1.64 × 10^−4^	1.44 × 10^−4^, 1.87 × 10^−4^		7 × 10 × 10^−5^	6.17 × 10^−5^, 8.08 × 10^−5^	
Year	6.90 × 10^−6^	5.40 × 10^−7^, 2.06 × 10^−5^		0.00	0.00, 0.00		1.03 × 10^−5^	8.80 × 10^−7^, 2.96 × 10^−5^		6.59 × 10^−5^	1.65 × 10^−5^, 1.68 × 10^−4^	
Residual	3.58 × 10^−3^	3.31 × 10^−3^, 3.81 × 10^−3^		7.72 × 10^−5^	7.19 × 10^−5^, 8.27 × 10^−5^		2.27 × 10^−3^	2.12 × 10^−3^, 2.43 × 10^−3^		1.39 × 10^−3^	1.30 × 10^−3^, 1.49 × 10^−3^	

Egg parameter	Volume			Pointedness			Elongation			Polar asymmetry		
Fixed effect	Mean	95% CI	*p*	Mean	95% CI	*p*	Mean	95% CI	*p*	Mean	95% CI	*p*
Intercept	**18.56**	**18.09, 19.03**	**< 0.001**	**5.73 × 10** ^**−1**^	**5.68 × 10** ^**−1**^, **5.78 × 10** ^**−1**^	**< 0.001**	**1.43**	**1.41, 1.45**	**< 0.001**	**2.32**	**2.17, 2.47**	**< 0.001**
Average age	**4.78 × 10** ^**−2**^	**1.23 × 10** ^**−2**^, **8.12 × 10** ^**−2**^	**0.006**	−3.17 × 10^−4^	−6.56 × 10^−4^, 2.87 × 10^−5^	0.065	**−2.33 × 10** ^**−3**^	**−3.88 × 10** ^**−3**^, **−8 × 10 × 10** ^**−4**^	**0.003**	−7.23 × 10^−3^	−1.69 × 10^−2^, 2.27 × 10^−3^	0.139
Delta age	**1.44 × 10** ^**−1**^	**1.56 × 10** ^**−2**^, **2.76 × 10** ^**−1**^	0.054	2.84 × 10^−4^	−8.18 × 10^−4^, 1.37 × 10^−3^	0.624	−2.58 × 10^−3^	−7.49 × 10^−3^, 2.25 × 10^−3^	0.361	2.48 × 10^−2^	−7.26 × 10^−3^, 5.64 × 10^−2^	0.163
CS.LO(2‐1)	2.29 × 10^−1^	−1.49 × 10^−1^, 6.03 × 10^−1^	0.232	−2.42 × 10^−3^	−6.63 × 10^−3^, 1.91 × 10^−3^	0.269	**−2.89 × 10** ^**−2**^	**−4.66 × 10** ^**−2**^, **−1.14 × 10** ^**−2**^	**0.001**	3.16 × 10^−2^	−1.08 × 10^−1^, 1.71 × 10^−1^	0.664
CS.LO(2‐2)	−3.27 × 10^−1^	−7.05 × 10^−1^, 5.93 × 10^−2^	0.088	**−6.19 × 10** ^**−3**^	**−1.04 × 10** ^**−2**^, **−1.90 × 10** ^**−3**^	**0.005**	**−3.24 × 10** ^**−2**^	**−5.02 × 10** ^**−2**^, **−1.52 × 10** ^**−2**^	**< 0.001**	−1.15 × 10^−2^	−1.52 × 10^−2^, 1.24 × 10^−1^	0.875
CS.LO(3‐1)	**4.16 × 10** ^**−1**^	**3.76 × 10** ^**−2**^, **7.94 × 10** ^**−1**^	**0.029**	−1.07 × 10^−3^	−5.39 × 10^−3^, 3.18 × 10^−3^	0.621	**−3.25 × 10** ^**−2**^	**−4.99 × 10** ^**−2**^, **−1.52 × 10** ^**−2**^	**< 0.001**	8.01 × 10^−2^	−5.93 × 10^−2^, 2.23 × 10^−1^	0.268
CS.LO(3‐2)	1.68 × 10^−2^	−3.56 × 10^−1^, 3.98 × 10^−1^	0.930	−2.51 × 10^−3^	−6.75 × 10^−3^, 1.84 × 10^−3^	0.246	**−4.62 × 10** ^**−2**^	**−6.36 × 10** ^**−2**^, **−2.88 × 10** ^**−2**^	**< 0.001**	6.05 × 10^−2^	−7.73 × 10^−2^, 2.01 × 10^−1^	0.401
CS.LO(3‐3)	**−6.30 × 10** ^**−1**^	**−1.01, −2.56 × 10** ^**−1**^	**0.001**	**−1.24 × 10** ^**−2**^	**−1.65 × 10** ^**−2**^, **−8.08 × 10** ^**−3**^	**< 0.001**	**−1.76 × 10** ^**−2**^	**−3.51 × 10** ^**−2**^, **−1.90 × 10** ^**−4**^	**0.042**	**−1.97 × 10** ^**−1**^	**−3.34 × 10** ^**−1**^, **−6.25 × 10** ^**−2**^	**0.006**
Difference between average age and delta age	9.66 × 10^−2^	−4 × 10 × 10^−2^, 3.36 × 10^−1^		5.98 × 10^−4^	−5.61 × 10^−4^, 1.76 × 10^−3^		−3.18 × 10^−4^	−5.64 × 10^−3^, 4.92 × 10^−3^		3.18 × 10^−2^	−1.18 × 10^−3^, 6.60 × 10^−2^	

Random effect	Mean	95% CI		Mean	95% CI		Mean	95% CI		Mean	95% CI	
Female identity	1.81	1.58, 2.06		1.56 × 10^−4^	1.35 × 10^−4^, 1.79 × 10^−4^		3.43 × 10^−3^	2.98 × 10^−3^, 3.94 × 10^−3^		1.15 × 10^−1^	9.64 × 10^−2^, 1.28 × 10^−1^	
Clutch identity	2.80 × 10^−2^	2.44 × 10^−2^, 3.20 × 10^−2^		5.56 × 10^−6^	4.85 × 10^−6^, 6.30 × 10^−6^		1.01 × 10^−4^	8.78 × 10^−5^, 1.14 × 10^−4^		2.02 × 10^−3^	1.76 × 10^−3^, 2.30 × 10^−3^	
Year	3.74 × 10^−2^	4.05 × 10^−3^, 1.06 × 10^−1^		1.31 × 10^−6^	2.09 × 10^−7^, 3.24 × 10^−6^		3.36 × 10^−5^	5.82 × 10^−6^, 8.81 × 10^−5^		7.92 × 10^−4^	8.89 × 10^−5^, 2.07 × 10^−3^	
Residual	7 × 10 × 10^−1^	6.62 × 10^−1^, 7.61 × 10^−1^		9.53 × 10^−5^	8.88 × 10^−5^, 1.02 × 10^−4^		1.46 × 10^−3^	1.36 × 10^−3^, 1.57 × 10^−3^		1.20 × 10^−1^	1.12 × 10^−1^, 1.29 × 10^−1^	

*Note:* Random effects included in the models were female identity, clutch identity (nested in female identity), and year. The table presents posterior mean estimates and associated 95% credible intervals (95% CI) for fixed and random effects. Significant fixed effects (*p* value < 0.05 and a 95% CI that does not overlap with zero) are presented in bold.

**FIGURE 4:**
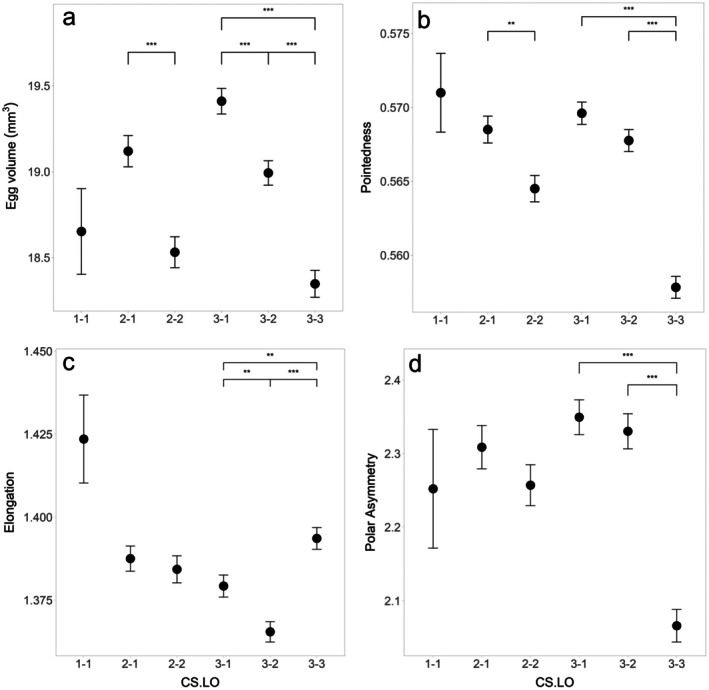
(a) Egg volume, (b) pointedness, (c) elongation, and (d) polar asymmetry as a function of the combination of clutch size (CS) and laying order (LO) assessed using 1589 eggs from 687 clutches laid by 330 individual female common terns in the years 2017–2020. Circles represent the means of the egg shape characteristics for each CS‐LO category, bars represent the standard errors. Significant differences between CS‐LO categories are indicated by the lines and stars at the top of the plots (**p < 0.01, ***p < 0.001).

We did not find any differences in shell spottiness and colour between eggs of a different laying order in clutches of a different size (all *p* values of CS.LO > 0.05 in ANOVA tests). For egg shape/size parameters, however, we did find such an effect (all *p* values of “CS.LO” < 0.001 in ANOVA tests): later laid eggs from two‐ and three‐egg clutches were smaller (Figure 4a) and less pointy (Figure 4b) with eggs in three‐egg clutches also becoming less asymmetric (Figure 4d) over the laying sequence. Finally, the second eggs from three‐egg clutches were less elongated than the first and the third eggs (Figure 4c).

## 4 Discussion

Birds are unique amniotes in the sense that they have evolved huge among‐ and within‐species variation in the colour, maculation, shape, and size of their eggs. In this study, performed on common terns across four breeding seasons, we found that all eight egg characteristics that we quantified were moderately to highly repeatable between and within clutches of the same female, and organized along two main axes of variation: one describing the colour and spottiness of egg shells, and one describing the size and shape of eggs. While some characteristics changed with age within females (egg volume and spottiness), others did not (colour variables and shape variables). Similarly, some egg characteristics varied with the laying order of eggs within a clutch (size and shape variables), while others did not (spottiness and colour variables). As such, we show that different egg characteristics are differently related to important life‐history variables, hinting at differences in adaptive significance among them.

The result that all egg characteristics that we measured were moderately to highly repeatable across females, regardless of whether they were measurements of eggs within clutches, or from clutches produced in different years, shows that females consistently produce eggs of a distinct morphology and suggests that these characteristics are not purely stochastic or environmental by‐products that form during the production of the egg and its shell. Such individual consistency has also been found in other studies (Beech et al. 2022; Dearborn et al. 2012; Hauber et al. 2019; Sulc et al. 2022), and may be mechanistically explained by a strong genetic basis of the process that creates these unique “signatures” (Gosler et al. 2000; Guo et al. 2020; Morales et al. 2010). From an adaptive point of view, this consistency could facilitate egg recognition and reduce the risk of brood parasitism (Fernandez‐Duque et al. 2025; Li et al. 2020). However, personal observations in the study colony taught us that incubating common terns readily accept any fake egg that roughly resembles a real egg (Figure S3), and maintain incubation when their real eggs are temporarily artificially incubated (e.g., Vedder, Kuerten, Bouwhuis, et al. 2017; Kürten et al. 2021). Alternatively, egg camouflage may favor the production of very similar‐looking eggs within a nest to avoid predator attraction (Stoddard et al. 2011; Westmoreland 2008; Westmoreland and Kiltie 2007), without narrowing down the large and consistent differences between females in egg characteristics that we observed. Indeed, experimental work on corvids suggests that their “hunting by searching image” can simultaneously select for crypsis and polymorphism, with polymorphism in prey appearance being maintained by frequency‐dependent selection (Bond and Kamil 2002). Perhaps, the large variation in egg characteristics between female common terns serves a similar function; the absence of a single egg phenotype among clutches in the population may make it more difficult for visual predators to develop an efficient searching image. Further research should thus focus on identifying the agents of selection on egg characteristics and quantifying how they act.

Some of the variables that determine egg characteristics are expected to be costly for the female and therefore to be related to female “quality” (Morales et al. 2006; Moreno and Osorno 2003; Reynolds et al. 2009). As such, we expected to find egg characteristics to vary with age, either based on the general increase in reproductive performance with age within females in our study species (Nisbet et al. 2020; Vedder et al. 2021; Zhang et al. 2015a, 2015b), or due to an association with lifespan, causing the egg characteristics of high‐quality females to be overrepresented among older females (Vedder and Bouwhuis 2018). The majority of the egg characteristics we measured (6 of 8), however, did not show any age specificity. We did find that eggshell spottiness increased with age within individual females, while at the same time finding that females producing spottier eggs seemed less represented among the females of older age classes, suggesting their selective disappearance from the breeding population. Following the hypothesis that spots reflect a female's oxidative level, as they are mainly due to the pigment protoporphyrin that can act as a prooxidant (Martinez et al. 2003; Martínez‐de la Puente et al. 2007), our result may suggest that oxidative stress increases with age within females and that females with high oxidative stress in early life, reflected in their spottier eggs (as observed for instance in great tits— *Parus major* —in Hargitai, Nagy, Herényi, et al. 2016), do not reproduce at older ages. Our study, however, is correlative and prevents us from inferring a causal relationship and pinpointing which female characteristics that change with age are responsible for the covariance between spottiness and female age. Further studies would be needed, and, for example, test for an association between a female's eggshell spottiness and her oxidative status and immune capacities.

Similar to previous studies in common terns (Gonzalez‐Solis et al. 2004; Zhang et al. 2015a; Nisbet et al. 2020), we found that egg volume increased with age within females. An increased egg volume may be beneficial for chick survival, as egg volume is a strong determinant of chick mass at hatching, which can have a carry‐over effect on chick fitness later on (Nisbet 1978; Reid and Boersma 1990; Williams 1994; Krist 2011; Vedder et al. 2023). Given these benefits, the increase in egg volume with age suggests that young females have a reduced ability to invest fully in eggs, and that this ability improves with age within individuals. Such improvement with age is also reflected in the general improvement of reproductive success with age in this species (Nisbet et al. 2020; Vedder et al. 2021; Zhang et al. 2015a, 2015b) and most likely results from increased experience in foraging, or better knowledge on local food availability (Forslund and Pärt 1995).

Within clutches, egg volume, as well as the three egg shape characteristics (pointedness, elongation, and polar asymmetry) decreased with laying order. This suggests these traits are linked (also see Birkhead 2016; Stoddard et al. 2017), and that the well‐known reduction of egg size with laying order in the common tern (Bollinger 1994; Garcia et al. 2011; Vedder, Zhang, and Bouwhuis 2017) is, thus, at least partly caused by a modification of egg shape toward less pointy, less long and less asymmetric eggs. In passerines, a reduction in egg volume with laying order is often attributed to a lack of calcium in females (Gosler et al. 2005; Graveland and Berends 1997; Graveland and Drent 1997). However, thanks to their piscivorous diet, seabirds should not suffer from calcium limitations (for common terns see Becker and Ludwigs 2004), such that the observed reduction in egg volume with laying order has more likely evolved as an adaptive strategy to facilitate an efficient “brood reduction strategy” when food availability during chick rearing turns out lower than anticipated during clutch formation (Slagsvold et al. 1984). Indeed, common terns typically produce an excess of chicks, of which the last‐hatched chicks rapidly die after hatching when food availability is low (Vedder et al. 2021; Vedder et al. 2019; Vedder, Zhang, and Bouwhuis 2017).

In sum, we found that egg characteristics were moderately to highly repeatable between female common terns, both within clutches, and among clutches laid in different years. Yet, specific aspects of egg characteristics were variably related to different aspects of the common tern's general life history, and may thus result from different selective pressures. We suggest further studies to identify the specific agents of selection that determine the adaptive function of the specific egg variables, to fully understand the eco‐evolutionary significance of this extended phenotype of the female.

## Author Contributions

**Coraline Bichet:** conceptualization (equal), data curation (lead), formal analysis (lead), methodology (lead), writing – original draft (lead). **Maria Moiron:** conceptualization (equal), formal analysis (supporting), methodology (supporting), writing – original draft (supporting). **Nathalie Kürten:** data curation (supporting), methodology (supporting), project administration (supporting), writing – original draft (supporting). **Oscar Vedder:** conceptualization (equal), formal analysis (supporting), methodology (supporting), writing – original draft (supporting). **Sandra Bouwhuis:** conceptualization (equal), formal analysis (supporting), funding acquisition (lead), methodology (supporting), project administration (lead), supervision (lead), writing – original draft (supporting).

## Conflicts of Interest

The authors declare no conflicts of interest.

## Supporting information


**Figure S1:** Intra‐specific variability in egg characteristics in our common tern colony.
**Figure S2:** Correlation matrix for eight egg characteristics assessed for 1589 common tern eggs from 687 clutches laid by 330 females in the years 2017–2020.
**Figure S3:** Common tern nest containing two fake eggs and one original egg. Despite the fake eggs roughly resembling the real egg, common terns do not hesitate to incubate the clutch, neither when only a single original egg is replaced by a fake egg, nor when the whole clutch is replaced.
**Table S1:** Estimates of the repeatability of measurements of eight egg characteristics obtained by analysing two pictures taken of different sides of a subset of 1430 eggs.
**Table S2:** Proportion and cumulative proportion of variance explained by principal component axes loading variation in eight egg characteristics (spottiness, Hue, Saturation, Value, volume, pointedness, elongation, and polar asymmetry) assessed using 3019 pictures of 1589 eggs from 687 clutches laid by 330 female common terns in the years 2017–2020.
**Table S3:** Factor loadings for eight egg characteristics assessed for 1589 eggs from 687 clutches laid by 330 female common terns in the years 2017–2020 onto the first two axes of the principal component analysis described in Table S2.
**Table S4:** Estimates of the repeatability of eight egg characteristics for female common terns (a) across years and (b) within clutches, assessed using 1589 eggs laid in 687 clutches by 330 individual female common terns in the years 2017–2020.

## Data Availability

Our data is available from the Dryad Digital Repository (DOI: https://doi.org/10.5061/dryad.6m905qgdk).
