# Supplementary material for: Egg Characteristics of Female Common Terns Are Repeatable, and Vary With Maternal Age and Laying Order
Source: Ecol Evol. 2025 Nov 19;15(11):e72455. doi: 10.1002/ece3.72455 (PMC12628015; doi:10.1002/ece3.72455)
Supplement: Supplementary file 1 — Figure S1: Intra‐specific variability in egg characteristics in our common tern colony. Figure S2: Correlation matrix for eight egg characteristics assessed for 1589 common tern eggs from 687 clutches laid by 330 females in the years 2017–2020. Figure S3: Common tern nest containing two fake eggs and one original egg. Despite the fake eggs roughly resembling the real egg, common terns do not hesitate to incubate the clutch, neither when only a single original egg is replaced by a fake egg, nor when the whole clutch is replaced. Table S1: Estimates of the repeatability of measurements of eight egg characteristics obtained by analysing two pictures taken of different sides of a subset of 1430 eggs. Table S2: Proportion and cumulative proportion of variance explained by principal component axes loading variation in eight egg characteristics (spottiness, Hue, Saturation, Value, volume, pointedness, elongation, and polar asymmetry) assessed using 3019 pictures of 1589 eggs from 687 clutches laid by 330 female common terns in the years 2017–2020. Table S3: Factor loadings for eight egg characteristics assessed for 1589 eggs from 687 clutches laid by 330 female common terns in the years 2017–2020 onto the first two axes of the principal component analysis described in Table S2. Table S4: Estimates of the repeatability of eight egg characteristics for female common terns (a) across years and (b) within clutches, assessed using 1589 eggs laid in 687 clutches by 330 individual female common terns in the years 2017–2020. [file ECE3-15-e72455-s001.zip › Bichet_et_al_Repeatability_eggs_SI_07102025.docx]

**SUPPLEMENTAL INFORMATION FOR**

**Egg characteristics of female common terns are repeatable, and vary with maternal age and laying order**

by

Coraline Bichet, Maria Moiron, Nathalie Kürten, Oscar Vedder and Sandra Bouwhuis

**Table S1:** Estimates of the repeatability of measurements of eight egg characteristics obtained by analysing two pictures taken of different sides of a subset of 1430 eggs. The table presents means and 95% credible intervals (95% CI).

| **Egg characteristic** | **Repeatability coefficient** | **95% CI** |
| --- | --- | --- |
| Spottiness | 0.909 | 0.901 – 0.917 |
| Hue | 0.983 | 0.981 – 0.984 |
| Saturation | 0.981 | 0.979 – 0.983 |
| Value | 0.957 | 0.953 – 0.961 |
| Volume | 0.990 | 0.989 – 0.991 |
| Pointedness | 0.971 | 0.968 – 0.974 |
| Elongation | 0.995 | 0.99 – 0.995 |
| Polar Asymmetry | 0.961 | 0.957 – 0.965 |

**Table S2:** Proportion and cumulative proportion of variance explained by Principal Component axes loading variation in eight egg characteristics (spottiness, Hue, Saturation, Value, volume, pointedness, elongation and polar asymmetry) assessed using 3019 pictures of 1589 eggs from 687 clutches laid by 330 female common terns in the years 2017 – 2020.

| **PCA axis** | **PC1** | **PC2** | **PC3** | **PC4** | **PC5** | **PC6** | **PC7** | **PC8** |
| --- | --- | --- | --- | --- | --- | --- | --- | --- |
| Proportion of variance | 0.346 | 0.215 | 0.136 | 0.110 | 0.096 | 0.049 | 0.034 | 0.014 |
| Cumulative proportion | 0.346 | 0.561 | 0.697 | 0.807 | 0.903 | 0.952 | 0.986 | 1.000 |

**Table S3:** Factor loadings for eight egg characteristics assessed for 1589 eggs from 687 clutches laid by 330 female common terns in the years 2017 – 2020 onto the first two axes of the Principal Component Analysis described in Table S2.

| **Egg characteristic** | **PC1** | **PC2** |
| --- | --- | --- |
| Spottiness | 0.458 | 0.087 |
| Hue | -0.468 | -0.015 |
| Saturation | 0.550 | 0.023 |
| Value | -0.504 | -0.076 |
| Volume | 0.059 | 0.073 |
| Pointedness | -0.055 | 0.673 |
| Elongation | -0.055 | 0.433 |
| Polar Asymmetry | -0.071 | 0.583 |

**Table S4:** Estimates of the repeatability of eight egg characteristics for female common terns (a) across years and (b) within clutches, assessed using 1589 eggs laid in 687 clutches by 330 individual female common terns in the years 2017 – 2020. The table presents means and 95% credible intervals (95% CI).

**a) Female repeatability across years**

| **Egg characteristic** | **Repeatability coefficient** | **95% CI** |
| --- | --- | --- |
| Spottiness | 0.665 | 0.632 – 0.695 |
| Hue | 0.725 | 0.696 – 0.751 |
| Saturation | 0.655 | 0.622 – 0.686 |
| Value | 0.654 | 0.617 – 0.688 |
| Volume | 0.699 | 0.665 – 0.730 |
| Pointedness | 0.604 | 0.568 – 0.636 |
| Elongation | 0.682 | 0.649 – 0.712 |
| Polar Asymmetry | 0.475 | 0.438 – 0.511 |

**b) Female within-clutch repeatability**

| **Egg characteristic** | **Repeatability coefficient** | **95% CI** |
| --- | --- | --- |
| Spottiness | 0.677 | 0.646 – 0.706 |
| Hue | 0.766 | 0.742 – 0.788 |
| Saturation | 0.678 | 0.648 – 0.707 |
| Value | 0.664 | 0.628 – 0.698 |
| Volume | 0.710 | 0.677 – 0.740 |
| Pointedness | 0.625 | 0.592 – 0.656 |
| Elongation | 0.702 | 0.672 – 0.730 |
| Polar Asymmetry | 0.483 | 0.447 – 0.519 |


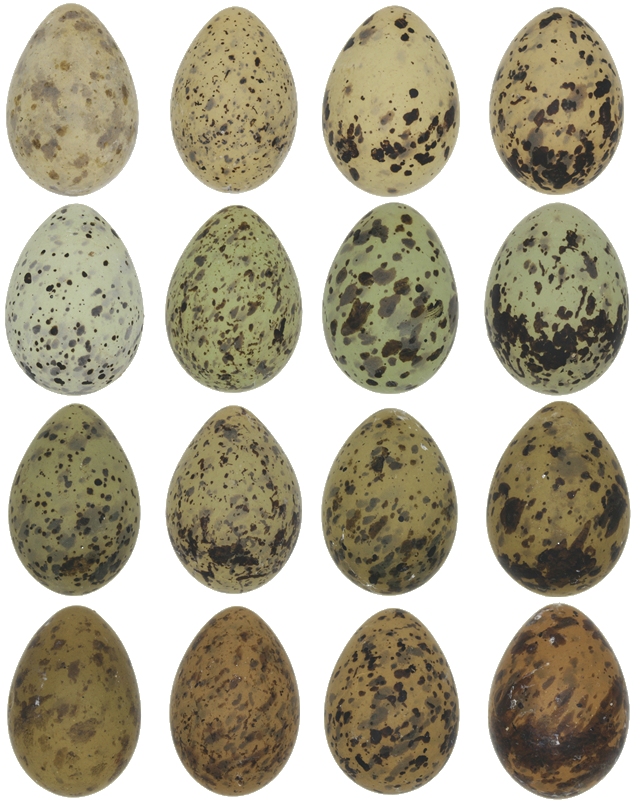


**Figure S1:** Pictures showing the intra-specific variability in egg characteristics in our common tern colony.

**Figure S2:** Correlation matrix for eight egg characteristics assessed for 1589 common tern eggs from 687 clutches laid by 330 females in the years 2017 – 2020. Positive correlations are displayed in blue, negative correlations in red, with the colour intensity proportional to the strength of the correlation coefficient. Crossed out coefficients indicate that the corresponding correlations are not significant (p-value > 0.05).

**-0.33**

**0.52**

**-0.84**

**-0.72**

**0.41**

**-0.66**

**0.08**

**-0.02**

**0.05**

**-0.06**

**0.02**

**0.06**

**-0.07**

**-0.03**

**0.07**

**-0.06**

**-0.03**

**-0.02**

**0.08**

**-0.09**

**0.37**

**-0.01**

**0.07**

**-0.07**

**0.04**

**0.05**

**0.54**

**0.13**

-1

-0.8

-0.6

-0.4

-0.2

0

0.2

0.4

0.6

0.8

1

Hue

Saturation

Value

Volume

Pointedness

Elongation

Polar Asymmetry

Spottiness

Hue

Saturation

Value

Volume

Pointedness

Elongation


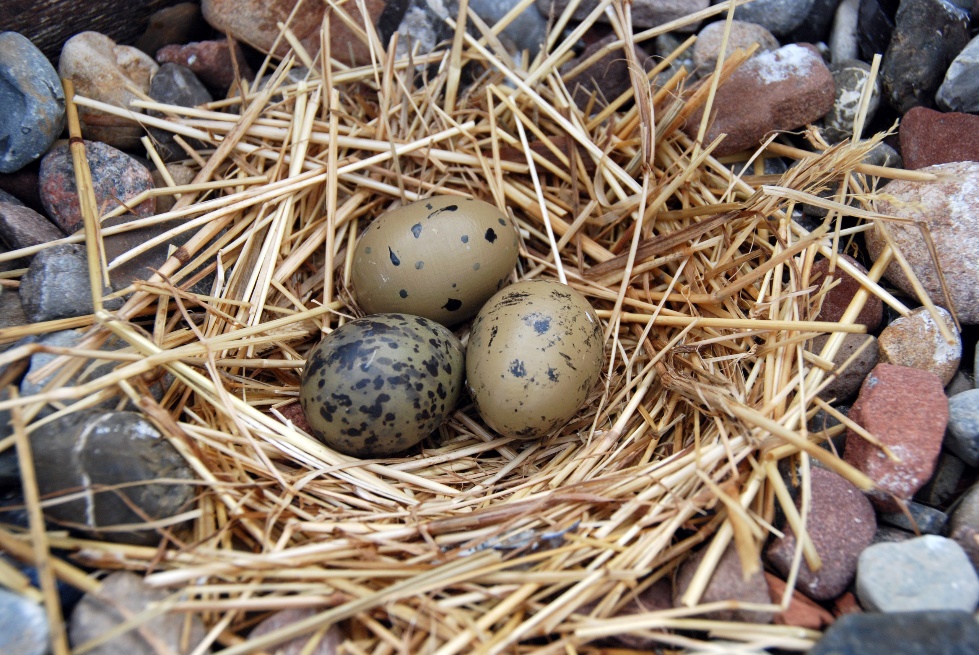


**Figure S3:** Picture of a common tern nest containing two fake eggs and one original egg. Despite the fake eggs roughly resembling the real egg, common terns do not hesitate to incubate the clutch, neither when one or two original eggs are replaced by fake eggs, nor when the whole clutch is replaced. photo: Sander Koezema.
